# Familial Aggregation in Vitamin D Deficiency Disorder

**DOI:** 10.7759/cureus.14685

**Published:** 2021-04-25

**Authors:** Leila R Farzin, Saeed Dastgiri

**Affiliations:** 1 Community and Family Medicine, Tabriz University of Medical Sciences, Tabriz, IRN; 2 Tabriz Health Services Management Research Centre, Tabriz University of Medical Sciences, Tabriz, IRN

**Keywords:** prevalence, vitamin d deficiency, familial aggregation, occurrence

## Abstract

Introduction

The relationship between vitamin D deficiency and some diseases (i.e., heart diseases, malignancies, and infectious diseases) has extensively been studied. There is, however, no epidemiological report on whether the familial predisposing factors have any role in the occurrence of vitamin D deficiency. The aim of this study was to investigate the familial aggregation of vitamin D deficiency disorder in the northwest region of Iran.

Materials and Methods

A total number of 930 individuals from the general population were invited/registered to the Family Medicine Clinic of Asadabadi Hospital, Tabriz University of Medical Sciences, Iran. A blood sample was obtained from subjects to measure the level of vitamin D. The blood tests were carried out by the enzyme-linked immunosorbent assay method using Biorexfars diagnostics in the Asadabadi Hospital Laboratory. We calculated odds ratio (OR) with 95% confidence intervals (95% CI) to estimate the aggregation of vitamin D deficiency among relatives.

Results

We ascertained 580 cases with vitamin D deficiency disorder representing an overall prevalence rate of 62.4% (95% CI: 59-65%) in the northwest region. An aggregation of the vitamin D deficiency was found among brothers (OR = 1.55, 95% CI: 0.72-3.32), sisters (OR = 1.24, 95% CI: 0.80-1.93), and spouses (OR = 1.18, 95% CI: 0.76-1.82) of the cases. Other relatives (including parents, grandparents, grandchildren, aunts, nieces, and nephews) showed no aggregation of deficiency in the family in this study.

Conclusion

Our findings indicated that there might be an aggregational occurrence of vitamin D deficiency in some of the family members. Therefore, to be able to perform early preventive intervention, we would suggest testing the blood level of vitamin D for brothers, sisters, and spouse if one was diagnosed as having vitamin D deficiency.

## Introduction

Vitamin D plays an important role in human health and meeting the biologic needs of human body. In the endocrine system, vitamin D controls the various parts of calcium hemostasis, especially in bone metabolism [1-3].

As a major public health problem, according to a systematic review and meta-analysis study, the overall prevalence of vitamin D deficiency was estimated to be 56% in the Iranian general population [4]. Another study found some degree of vitamin D deficiency in 84% of females and 68% of males among healthy children and adolescents living in the capital, Tehran [5]. A recent meta-analysis of 48 studies on 18,531 Iranian individuals estimated the prevalence of vitamin D deficiency for males (46%), females (62%), and pregnant women (60%) in various geographical regions in Iran [6].

Genetic epidemiology examines the impact of genetic factors, environmental exposures, and the interaction between these two elements on the occurrence of a disease or a trait, and that how and why diseases, traits, and the related causes cluster in certain groups. Familial aggregation, as part of the genetic epidemiology, is the study of a group of individuals with a specific disease or trait to identify whether relatives have an excess occurrence of the same disease or trait.

Little is known about the role of genetic and environmental factors, and the interaction in between, on the plasma level and deficiency of vitamin D. Wang and colleagues found a role for common genetic variants in regulation of circulating 25-hydroxy-vitamin D (25OHD) concentrations in a large group of 33,996 European descent individuals [7]. Another study on the 95 nuclear pedigrees in Russia showed that there was a very high pair-wise correlation between all members of families for 25OHD, indicating that there might be a genetic factor transmitted within those families for 25OHD measures [8]. Similar evidence was found in school-aged children and their parents in nine Mesoamerican countries where the family aggregation of 25OHD concentrations was high among mothers and children [9].

The aim of this study was to investigate the familial aggregation of vitamin D deficiency disorder in the northwest region of Iran.

## Materials and methods

A total number of 930 individuals from the general population were invited/registered to the Family Medicine Clinic of Asadabadi Hospital, Tabriz University of Medical Sciences, Iran. We recruited 369 participants plus 561 of their family members, including brothers, sisters, spouses, mothers, fathers, grandmothers, grandfathers, grandchildren, aunts, and nephews for the assessment of vitamin D familial aggregation as hereditary, or as having a common lifestyle environment at home (i.e., husband and wife as spouses). As the inclusion and exclusion criteria for this study, none of the study subjects had a certain disease, did not use any particular medication, and gave informed consent for participating in this study. The blood samples were obtained from the study subjects in the morning following overnight fasting to measure the level of vitamin D. Blood tests were carried out by the enzyme-linked immunosorbent assay method (Awareness Technology Stat Fax 4200) using Biorexfars diagnostics (www.biorexfars.com) in the Asadabadi Hospital Laboratory. Based on the plasma level of the vitamin D in the study subjects, all amount less than 25 nanograms per milliliter (ng/ml) was considered as the deficiency of vitamin D in this study.

We calculated odds ratio (OR) with 95% confidence intervals (95% CI) to measure the aggregation of vitamin D deficiency among relatives. The prevalence rate including 95% CI was also estimated for the study population.

## Results

The study participants (n = 930) comprised 606 (65.2%) female and 324 (34.8%) male subjects recruited in Asadabadi Hospital. The mean ± standard deviation age was 39.6 ± 18.1 with a median of 39 years.

We ascertained 580 cases with vitamin D deficiency disorder representing an overall prevalence of 62.4% (95% CI: 59-65%) in the northwest of the region. The prevalence rates were 60.9% (95% CI: 57.0-64.8%) and 63.6% (95% CI: 58.3-68.8%) in females and males, respectively.

**Figure 1:**
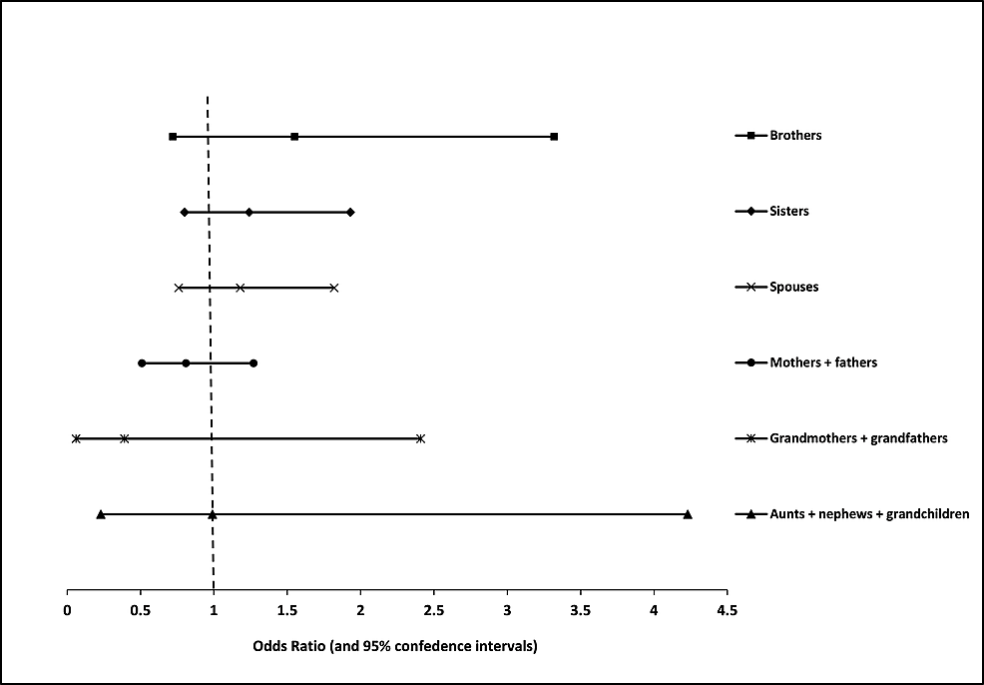
Familial aggregation of vitamin D deficiency in the family members and relatives

Figure 1 presents the familial aggregation of vitamin D deficiency in the family members and relatives of the study subjects. An aggregation of the vitamin D deficiency was found among brothers (OR = 1.55, 95% CI: 0.72-3.32), sisters (OR = 1.24, 95% CI: 0.80-1.93), and spouses (OR = 1.18, 95% CI: 0.76-1.82) of the cases. Other relatives (including parents, grandparents, grandchildren, aunts, nieces, and nephews) showed no aggregation of deficiency of vitamin D in this study.

## Discussion

In this cross-sectional study, the prevalence and familial aggregation of vitamin D deficiency was investigated in a northwest region of Iran.

Very little is known about the occurrence of familial aggregation for chronic disorders in Iran. An aggregation of the risk factors of cardiovascular diseases has been reported from Kerman city in southern Iran [9]. We found that there is a familial aggregation of vitamin D deficiency among sisters, brothers, and spouses. Robinson et al. showed the familial aggregation for the same disorder among parents and children while we did not find the similar pattern in those family members [10].

This study showed that the occurrence of vitamin D deficiency is 61% and 64% in males and females, respectively. According to a systematic review in Iran, the prevalence of the deficiency of vitamin D was 62% in women and 46% in men [6]. The high occurrence of this disorder in various geographical areas in Iran indicates that this health problem needs to be considered as a priority of the healthcare system in the country, and a matter of large-scale etiological and preventive researches.

In a study conducted by Arora et al. in patients with prehypertension in the United States, 73% had vitamin D deficiency where the serum level of vitamin D in these patients was 15.7 ± 6.3 ng/ml [11]. Almost consistent with the above study, 62% of our subjects had deficiency with 18.4 ± 7.5 ng/ml of the serum level of vitamin D.

As for the limitations of this study, we did not have access to some socio-demographic and background data for statistical analysis of the occupation of participants, and the time duration of exposing to sunlight, as important factors in the occurrence of vitamin D deficiency. For the similar reason, we did not analyze the other potential factors related to the familial aggregation observed among the study participants to discuss whether they live together in one place under the same condition, or they have separate living with different lifestyle. Further investigations may then be required to clarify the independent role of genetics and environmental factors, and the interaction in between for the assessment of vitamin D deficiency disorder.

## Conclusions

Although there is still no clear evidence about the role of genetic and environmental factors on the occurrence of vitamin D deficiency, our findings indicated that there might be an aggregational pattern of vitamin D deficiency in some of the family members. Therefore, to be able to perform early preventive intervention, we would suggest testing the blood level of vitamin D for brothers, sisters, and spouse if one was diagnosed as having vitamin D deficiency.
